# Impact of Improving Quality of Dialysis Fluid on Oxidative Stress and Lipid Profile in Hemodialysis Patients

**DOI:** 10.5402/2013/717849

**Published:** 2012-12-19

**Authors:** Driss Elkabbaj, Abdelali Bahadi, Yahia Cherrah, Mourad Errasfa, Rachid Eljaoudi

**Affiliations:** ^1^Nephrology Department, Military Hospital Mohammed V, 10000 Rabat, Morocco; ^2^Pharmacology and Toxicology Department, Faculty of Medicine and Pharmacy, 10000 Rabat, Morocco; ^3^Pharmacology Department, Faculty of Medicine and Pharmacy, 30000 Fes, Morocco

## Abstract

The aim of this study was to evaluate the levels of malondialdehyde as an oxidative stress marker in the same hemodialysis patients after changing the quality of dialysate with ultrapure dialysis fluid. *Methods*. This prospective study concerns hemodialysis patients; all patients were in the first step treated with conventional dialysate, and in the second step (three months later) the same patients were treated with online produced ultrapure dialysis fluid. The malondialdehyde, C-reactive protein, total cholesterol, triglycerides, high-density lipoprotein, low-density lipoprotein, fibrinogen, and albumin were quantified before the two steps. *Results*. Thirty-seven patients completed the study. Ultrapure dialysis fluid reduced but not significantly the malondialdehyde concentrations. Both dialysis fluids were associated with improvement in the malondialdehyde level before and after the hemodialysis session. In lipid parameters, there was a significant decrease with conventional dialysis fluid versus ultrapure dialysis fluid of triglycerides, total cholesterol, and high-density lipoprotein in patients' blood. Instead, the level of low-density lipoprotein, fibrinogen, albumin, and C-reactive protein does not change significantly. *Conclusion*. The lipid parameters were improved for triglycerides and total cholesterol. Malondialdehyde increases following the hemodialysis session, and the conventional dialysate increased malondialdehyde levels more than the ultrapure dialysis but the differences were not statistically significant.

## 1. Introduction

The chlorine compounds used to suppress bacterial growth in the potable water supply are removed when the water is treated for hemodialysis. It is almost impossible to completely prevent bacterial proliferation in the treated water and the dialysate. As a result, even though conventional dialysate meets the required quality standards, it usually contains some low level of microbiological contamination, including fragments of endotoxin and peptidoglycans and bacterial fragments [1–3]. These contaminants, sometimes collectively referred to as “cytokine-inducing substances,” cross both low-flux and high-flux hemodialysis membranes [2, 4] and stimulate cytokine production by inflammatory cells [5]. The use of dialysate of much higher microbiological purity improved this state of inflammation [6, 7]. General markers of inflammation such as serum C-reactive protein (CRP), ferritin, or fibrinogen are commonly used, but the oxidative stress referred to excessive production of reactive oxygen species (ROS) and inadequate antioxidant protection, is more sensitive, specific, and precocious of inflammation state. This condition leads to structural and/or functional deterioration in cell components including DNA, proteins, carbohydrates, and lipids [8]. The presence of ROS can cause damage in many molecules, such as lipids, leading to the production of malondialdehyde (MDA), an indicator of lipid peroxidation [9–11]. In chronic renal failure (CRF) patients under hemodialysis (HD) treatment, the formation of ROS is amplified, therefore beyond uremic toxins [12, 13]. The aim of this study was to evaluate the oxidative stress using quantification of MDA in the CRF patients after changing quality of dialysate with ultrapure dialysis fluid.

## 2. Materials and Methods

Concerned patients were with end-stage renal disease (ESRD) on maintenance hemodialysis. Inclusion criteria were hemodialysis patients for at least 6 months. Exclusion criteria were (i) chronic infection; (ii) chronic inflammatory disorders; (iii) primary or secondary hyperlipidemia (other than uremic); (iv) major comorbid conditions such as severe heart failure, severe chronic obstructive lung disease, liver cirrhosis, or malignancy; and (v) unwillingness to participate in the study.

### 2.1. Study Design

This prospective unicenter study was conducted according to the principles of the Declaration of Helsinki and was approved by the local Ethical Committee from the Faculty of Medicine and Pharmacy in Rabat, Morocco. 

Patients were in the first step treated with conventional dialysate (double osmosis, deionization, and carbon filtration); in the second step (three months later) they were treated with online produced ultrapure dialysis fluid (Diasafe and heat disinfection with hotfeed Fresenius Medical Care, with reverse osmosis, deionization, and carbon filtration) during three months. Before starting, we analyzed the microbiological quality of dialysis fluid in the two water treatments. Hemodialysis sessions were performed with the same condition using bicarbonate as a buffer. All patients received single-use biocompatible synthetic low-flux membranes (Polyamide, Polyflux Renal Products Gambro). Blood flow rates were chosen between 300 and 350 mL/min, and ultrafiltration rates were set according to individual needs. Dialysate flow rate was fixed at 500 mL/min. In all the patients, the vascular access was arteriovenous fistula. For all treatments, heparinization of the individual patient did not differ throughout the study period. Hemodialysis was prescribed and monitored using a single pool urea kinetic model to ensure a delivered dialysis dose of at least 1.2 per dialysis for thrice weekly sessions.

### 2.2. Study Parameters

#### 2.2.1. Blood Sample

For MDA analysis, samples were collected in EDTA-containing tubes just before commencing dialysis (pre-HD) and at the termination of dialysis procedure (post-HD). Blood venous samples (10 mL) were centrifuged at 1500 ×g for 10 min just after being collected. The resulting plasma samples were frozen at −80°C until analysis. Serums were used to perform analysis of CRP, total cholesterol, TG, high-density lipoprotein (HDL), low-density lipoprotein (LDL), fibrinogen, and albumin before the two steps.

#### 2.2.2. Microbiological Quality of Dialysis Fluid

Sample volumes were 500 mL for both purified water and ultrapure dialysis fluid. We applied a membrane filtration technique for the ultrapure dialysis fluid cultures, using a microfilter with pore size 45 *μ*m (22–45). Tryptone glucose extract agar media were used for bacterial culture. Limulus amebocyte lysate (LAL) assay was used for determination of endotoxins (chromogenic method) according to the manufacturer [14].

#### 2.2.3. Physiological Parameters

**Figure 1:**
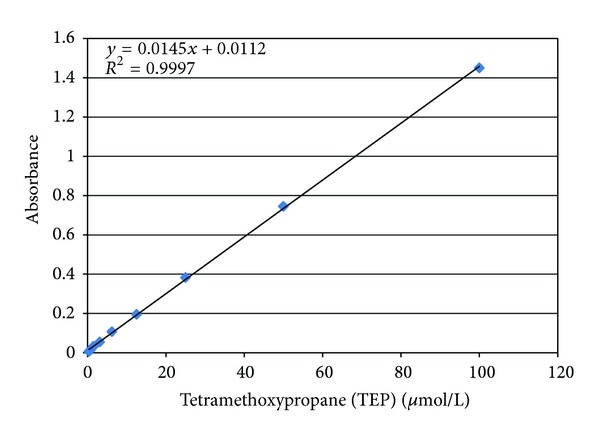
MDA calibration curve.

Serum total cholesterol, triglyceride, HDL, LDL, and CRP levels were determined using Dimension RXL Analyser (Dade Behring, Inc). MDA was determined by Thiobarbituric Acid Reactive Substances (TBARS) method. All chemicals and reagents used (from Merck) were of analytical grade, and Milli-Q water was used for each dilution. The analysis was performed according to published procedures for preparing MDA adduct [15, 16]. Briefly, a plasma sample (100 *μ*L) was mixed to 300 *μ*L of a 42 mM thiobarbituric acid (42 mM) solution and 700 *μ*L of a phosphoric acid solution (1%). The whole volume was incubated in a water bath at 95°C for 45 minutes. The reaction was then stopped at ice cold temperature, and an equal volume of n-Butanol was added to the reaction mixture. Samples were then centrifuged and an aliquot of the supernatant was read at 532 nm. A calibration curve was prepared with TEP (1,1,3,3-tetramethoxypropane) as standard MDA of 0.38 up to 100 *μ*mol/L. A linear regression between TEP concentration and absorbance was constructed using the Microsoft Excel software for Widows, and the regression equation was used to calculate the MDA concentration in each sample. In our study, we have taken into account only the calibration curves which had a coefficient of determination (*r* ^2^) more than 0.99 (Figure 1). Repeatability was confirmed by corresponding coefficient of variation of 4.11%. Recovery values of 97% indicated adequate accuracy of the method. During the measurement, each sample was analyzed in duplicate. These analyses were performed on the same patients in similar conditions and manner when using conventional dialysate and three months later after switching to ultrapure dialysate.

#### 2.2.4. Statistical Analysis

Data are presented as mean ± standard deviation (SD), median and interquartile, or as a percentage. According to normal or nonnormal distribution of data, comparison between variables is performed using the *t*-test, Mann-Whitney *U* test, Wilcoxon's test, chi square or Fisher's exact test. If applicable, an analysis of variance was used. Pearson's or Spearman's correlation was performed to determine the relationship between variables. Multivariable linear or logistic regression analysis was conducted to investigate independent determinants of TBARS. Differences were considered to be statistically significant if the *P* values were <0.05. All analyses were performed using the SPSS 13.0 for Windows (SPSS, Inc., Chicago, IL, USA).

## 3. Results

Thirty-nine patients were screening, and thirty-seven completed the study. Two patients were excluded for chronic inflammatory (one had malignancy disease, and the other had chronic infection on his foot). They were in hemodialysis for at least 24 months. The characteristics of these patients are given in Table 1. Nineteen males and eighteen females were with mean age of 50.7 ± 16.55 years. The causes of ESRD were diabetic nephropathy (15%), chronic glomerulonephritis (20%), chronic interstitial nephritis (17.5%), and unknown (32.5%). The microbiological parameters of dialysis fluid in the two water treatments of our center are presented in Table 2. 

Blood lipid analyses of patients were performed before and three months after that switching from conventional to ultrapure dialysis fluid. Our data show significant changes in blood lipid have occurred upon the above switching (Table 3). There was a significant decrease of TG (1.38 ± 0.62 versus 1.13 ± 0.37; *P* = 0.006), total cholesterol (1.72 ± 0.44 versus 1.38 ± 0.13; *P* = 0.001), and HDL (0.39 ± 0.10 versus 0.32 ± 0.12; *P* = 0.02) in patients' blood. Instead, the level of LDL, fibrinogen, albumin, and CRP did not change significantly. 

The use of ultrapure dialysis fluid reduced but not significantly the oxidative stress as evidenced by reduction in MDA concentrations (Tables 4 and 5). Both dialysis fluids were associated with improvement of the MDA level before and after HD session with *P* < 0.001 (Table 4). In multivariate study we found no statistically significant correlation between the value of MDA and other parameters (CRP, TG, total Cholesterol, LDL, HDL, fibrinogen, and albumin). We found also that causes of ESRD did not affect changes in values of MDA.

## 4. Discussion

In this study, we confirm that MDA increases in blood's patient following HD session, and we found that the conventional dialysate increased MDA levels more than ultrapure dialysate but the differences were not statistically significant. In multivariate study, it was shown that the MDA is a good marker for assessing oxidative stress generated by the water quality in HD because there is no influence of other inflammatory parameters. Effectively the European Best Practice Guidelines for Hemodialysis set the maximum allowable level for bacteria and endotoxin concentrations at 100 CFU/mL and 0.25 EU/mL, respectively [17]. For ultrapure dialysate, it is commonly defined as having a bacterial count less than 100 CFU/L and an endotoxin content less than 0.03 sEU/mL measured by the Limulus amebocyte lysate assay [18]. Our microbiological parameters of dialysis fluid were in agreement with these guidelines. HD patients are particularly vulnerable to contaminants in the water used to prepare concentrate and dialysate or in water used for reprocessing dialyzers. Compared to healthy individuals, HD patients are exposed to extremely large volumes of water having inadequate barriers to such toxins and cannot easily eliminate contaminants. The estimated water intake of a healthy individual is 2 liters per day or 14 liters per week. By comparison, HD patients may be exposed to 350 to 500 liters of water per week, depending on their treatment time and dialysate flow rate [19, 20]. With normal individuals, the gastrointestinal tract separates blood from contaminants in the water. By comparison, the barrier between blood and water in HD patients is a thin membrane through which the transfer of contaminants is limited only by the size of the contaminant. Schiffl and Lang demonstrate that inflammation, oxidative stress, and dyslipidemia are biologically linked [21]. This relation exists also in our study. On the other hand, the ultrapure dialysis fluid is associated with an improved cardiovascular risk factor profile [21]. This is the limit of our work since it is of short duration, which does not allow us to assess the impact of cardiovascular long term.The use of ultrapure dialysate produces a lesser degree of oxidative stress [22]. The lipid peroxidation is hallmark of oxidative stress, which disrupts the structural integrity of cell membranes and can also lead to the formation of aldehydes, which in turn by time damage lipids, proteins, and nucleic acid [23]. In addition, this state is involved in many pathophysiological processes, particularly within accelerated atherosclerosis, which is, at least in part, resistant to conventional pharmacotherapy [24], inflammation, and cancer [25, 26]. Switching from conventional dialysis fluid to ultrapure dialysis fluid has been shown repeatedly to be associated with a decrease in the circulating concentrations of biomarkers of inflammation [27, 28]. But not all studies report a reduction in inflammatory markers after the introduction of ultrapure dialysate [29, 30]. There are clearly many stimuli to inflammation in hemodialysis patients, such as the presence of synthetic grafts for blood access and the practice of dialyzer reuse. Thus, a failure to find a reduction in the level of inflammatory markers with ultrapure dialysate may reflect the presence of multiple inflammatory stimuli. Guo et al. find that contaminants in the bicarbonate and salt mixture used for preparation of dialysate are important factors for increased apoptosis in monocyte- like cell; in this study, the authors provide evidence of the association between an increased monocyte apoptosis rate and impurity of dialysate [31]. The oxidative stress could play a key role in this increased apoptosis rate as it participates in both the initiation and maintenance of the apoptotic process in monocytes stimulated by oxidants and proinflammatory cytokines [32]. Valentini et al. suggest that MDA levels suffer influence room time of HD treatment, as shown in our data.

## 5. Conclusion

Quantification of MDA allowed us to evaluate oxidative stress in hemodialysis patient. In this study, the lipid parameters were improved for TG and total cholesterol. We confirm that MDA increases in blood's patient following hemodialysis session on one hand and on the other hand we found that the conventional dialysate increased MDA levels more than ultrapure dialysis but the differences were not statistically significant.

## Figures and Tables

**Table 1:** Characteristics of patients.

Variable	Value
Age (years) m ± sd	50.7 ± 16.5
Sex ratio	19/18 (male/female)
Initial nephropathy: *n* (%)	
Undetermined nephropathy	15 (38)
Tubulointerstitial nephropathy	8 (22)
Diabetic	6 (16)
Glomerular nephropathy	6 (16)
Amyloidosis	1 (3)
Myeloma	1 (3)
Duration in hemodialysis (months) median (IQ, 25, 75)	36 (16.5–106)
Body mass index (Kg/m^2^) m ± sd	23.3 ± 3.4
Single pool *Kt*/*V* m ± sd	1.5 ± 0.19

**Table 2:** Microbiological parameters of dialysis fluids in the two water treatments.

Determination	Higher standards or limits (9.22)	Conventional dialysate	Ultrapure dialysate
Total germs—departure HD buckles (CFU/mL)	100	10	7
Total germs—return HD buckles (CFU/mL)	100	20	9
Endotoxin departure HD buckles (EU/mL)	0.03	0.01	<0.005
Endotoxin return HD buckles (EU/mL)	0.03	0.10	<0.005

**Table 3:** Blood lipids and other parameters before and after switching from conventional to ultrapure dialysis fluid.

Variable	Conventional dialysis fluid	Ultrapure dialysis fluid	*P*
Triglycerides (mg/L)	1.38 ± 0.62	1.13 ± 0.37	0.006*
Cholesterol (mg/L)	1.72 ± 0.44	1.38 ± 0.13	0.001*
LDL (mg/L)	1.01 ± 0.36	0.98 ± 0.30	0.6
HDL (mg/L)	0.39 ± 0.10	0.32 ± 0.12	0.02*
Fibrinogen (mg/L)	4.1 ± 0.86	4.0 ± 1.0	0.6
Albumin (g/L)	35.9 ± 2.3	35.0 ± 4.0	0.2
CRP (mg/L)	2.5 (0.15–7.2)	1.8 (0.1–7.4)	0.3

*Statistically significant.

**Table 4:** The values of MDA before and after hemodialysis session.

	MDA (*μ*Mol/L) before hemodialysis session	MDA (*μ*Mol/L) after hemodialysis session	*P*
Conventional dialysate	8.6 ± 1.5	13.0 ± 6.5	<0.001*
Ultrapure dialysate	7.9 ± 1.8	10.9 ± 3.5	<0.001*

*Statistically significant.

**Table 5:** The values of MDA before and after and MDA difference in conventional dialysate and ultrapure dialysate fluid.

	Conventional dialysate	Ultrapure dialysate	*P*
MDA1 (*μ*Mol/L) before hemodialysis session	8.6 ± 1.5	7.9 ± 1.8	0.12
MDA2 (*μ*Mol/L) after hemodialysis session	13.0 ± 6.5	10.9 ± 3.5	0.10
MDA difference (MDA2-MDA1)	4.6 ± 6.4	2.9 ± 2.9	0.2
